# Danaparoid sodium attenuates the increase in inflammatory cytokines and preserves organ function in endotoxemic rats

**DOI:** 10.1186/cc6943

**Published:** 2008-07-06

**Authors:** Toshiaki Iba, Taku Miyasho

**Affiliations:** 1 Department of Emergency and Disaster Medicine, Juntendo University, Tokyo, Japan, 2-1-1 Hongo, Bunkyo-ku, Tokyo 113-8421, Japan; 2 Department of Veterinary Biochemistry, School of Veterinary Medicine, Rakuno Gakuen University, 582 Bunkyodai-Midorimachi, Ebetsu, Hokkaido 069-8501, Japan

## Abstract

**Introduction:**

Anticoagulant therapy attracts much attention for the treatment of severe sepsis since recent studies have revealed that some anticoagulants have the ability to regulate the inflammatory response. The purpose of this study was to examine whether danaparoid sodium (DA) is effective for the treatment of organ dysfunction in sepsis.

**Methods:**

Sixty-four Wistar rats were intravenously injected with 5.0 mg/kg of lipopolysaccharide (LPS) and then divided into two groups: the DA group and the control group (n = 32 each). The DA group was injected intravenously with 400 U/kg of DA immediately after LPS injection, whereas the control group received saline. Blood samples were drawn at 1, 6, 12, and 24 hours after LPS injection, and organ damage markers and coagulation markers were measured. In the other series, 10 rats treated with LPS were divided into DA and control groups (n = 5 each). Blood samples were collected at 1, 3, and 6 hours after LPS injection and served for the cytokine measurements.

**Results:**

The elevation of the organ damage markers, such as alanine aminotransferase and lactate dehydrogenase, was significantly suppressed in the DA group. Coagulation markers, such as AT activity and fibrinogen levels, were maintained better in the DA group at 6 hours. The elevation of proinflammatory cytokines such as tumor necrosis factor-alpha, interleukin (IL)-1, and IL-6 was significantly suppressed in the DA group. On the other hand, there was no significant difference in anti-inflammatory cytokines such as IL-4 and IL-10.

**Conclusion:**

DA preserves the organ dysfunction in LPS-challenged rats. Although the mechanism is not fully elucidated, not only the improvement of coagulation disorder but also the regulation of circulating levels of proinflammatory cytokines may play a role in the mechanism.

## Introduction

Danaparoid sodium (DA) is a low-molecular-weight heparinoid with a mean molecular weight of approximately 6,000 daltons. It consists mainly of heparan sulfate (HS) (83%) and dermatan sulfate (12%). The high-affinity fraction of HS inhibits factor Xa by catalyzing its binding to antithrombin (AT) [1]. Recently, HS and syndecan, a major cell surface HS proteoglycan (HSPG), have attracted much attention as modulators of various types of inflammation since they have been known to bind and regulate many inflammatory factors, including inflammatory cytokines, through their HS chains [2-5]. Moreover, recent data indicate that HS and syndecan protect the host from various inflammatory disorders by neutralizing chemokines, attenuating exaggerated T-lymphocyte homing, and confining neutrophil migration to specific sites of tissue injury. Several studies have suggested that binding of chemokines to cell surface HS might regulate the cellular responses and migration of inflammatory cells [6]. HS can also function as a soluble molecule since the core protein to which it is covalently complexed can be released from the cell surface by proteolytic cleavage. Once solubilized, HS exhibits functions similar to or distinct from immobilized HS, and soluble HS will inhibit cell surface receptor-ligand interactions or it can alter the conformation of ligands or receptors to potentiate or attenuate their activities [7]. For example, soluble HS binds and potentiates activities of transforming growth factor-beta [8] and matrix metalloproteinase [9]. From these data, syndecan and its component HS are thought to act as key endogenous modulators of tissue injury and inflammation *in vivo*. In the present study, we hypothesized that externally administered HS can modulate the inflammatory reaction, and the primary purpose of this study was to examine the anti-inflammatory effects of HS in a sepsis model.

## Materials and methods

Ten-week-old Wistar rats were used in this study. All experimental procedures were conducted after obtaining the approval of the ethical committee for animal experiments of Juntendo University (Tokyo, Japan). All rats were provided standard rat chow and water *ad libitum*. The rats were anesthetized with sodium pentobarbital (40 mg/kg, intraperitoneally), and systemic inflammation was induced by administering a single injection of lipopolysaccharide (LPS) (*Escherichia coli *O55-B5; Difco Laboratories, Detroit, MI, USA) via the caudal vein at a dose of 5.0 mg/kg. In the first series, 64 animals were divided into two groups: the DA group (n = 32), in which intravenous administration of 400 U/kg of DA (Orgaran; Nippon Organon Co., Osaka, Japan) was performed immediately after LPS injection, and the control group (n = 32), in which animals were given equal volumes of saline (intravenously) immediately after the injection of LPS. One, 6, 12, and 24 hours after LPS injection, blood samples were obtained under anesthesia from the inferior vena cava, and samples served for the measurement of organ damage markers such as alanine aminotransferase (ALT), lactate dehydrogenase (LDH), and blood urea nitrogen (BUN). Coagulation markers, including AT activity, fibrin/fibrinogen degradation products (FDP), and fibrinogen levels and red blood cell (RBC), white blood cell (WBC), and the platelet counts, were measured in the same samples. Enzymatic activity of LDH was measured by an LDH-J kit (Wako Pure Chemical Industries, Osaka, Japan). BUN was measured by chemical colorimetric tests (UN-S; Seiken Chemical Industries Co., Ltd., Tokyo, Japan). AT activity was measured by chromogenic peptide substrate assay. Determinations of FDP and fibrinogen were performed by an enzyme-linked immunosorbent assay kit (Teikoku Laboratories, Tokyo, Japan). Blood cell count was calculated by an electric cell counter (Coulter Counter Model CBC5; Coulter Electronics, Bath, UK). In the second series, the same model was made (n = 10) and those rats were divided into DA and control groups (n = 5, each). In this series, blood samples were taken at 1, 3, and 6 hours after LPS injection. Tumor necrosis factor-alpha (TNF-α), interleukin (IL)-1α, IL-β, IL-2, IL-4, IL-6, IL-10, granulocyte-macrophage colony-stimulating factor (GM-CSF), and interferon-gamma (INF-γ) levels were measured using a Bio-Plex system (Rat Cytokine 9-Plex A Panel; Bio-Rad Laboratories, Inc., Hercules, CA, USA).

### Statistical analysis

All data are expressed as the mean ± standard deviation. A statistical analysis was performed using the Mann-Whitney *U *test with the Stat View II statistical software package for Macintosh. Statistical differences were deemed significant at less than 0.05.

## Results

**Figure 1:**
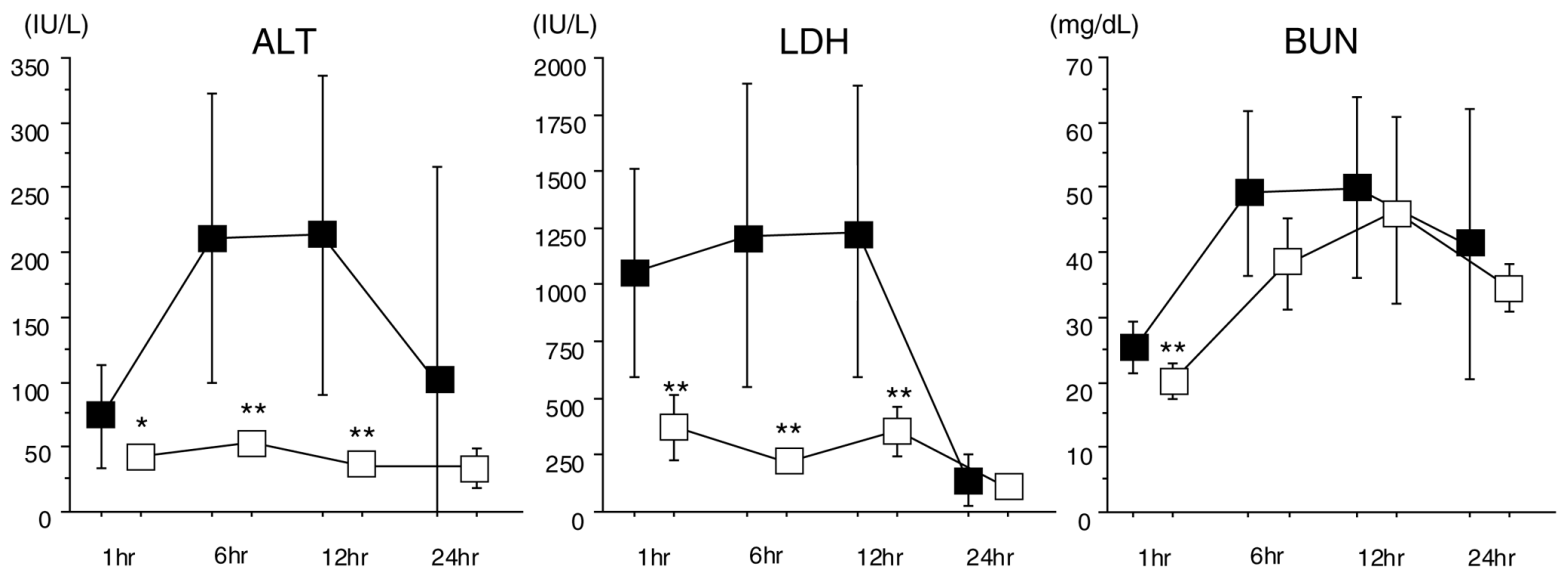
The changes in alanine aminotransferase (ALT), lactate dehydrogenase (LDH), and blood urea nitrogen (BUN) after lipopolysaccharide (LPS) injection. ALT increased significantly 6 hours after LPS injection in the control group but did not change in the danaparoid sodium (DA) group; the difference was significant from 1 to 12 hours after LPS injection. Similarly, significant LDH elevation is recognized from 1 to 12 hours after LPS injection in the control group. In contrast, the level stayed almost in the normal range during the experimental period in the DA group. There was no significant difference in the peak BUN level, but the difference was significant 1 hour after LPS injection (*P < 0.05, **P < 0.01) (■: control group, □: DA group, n = 8 in each group).

ALT increased significantly from 6 to 12 hours after LPS injection in the control group, whereas the elevation stayed in the lower level in the DA group (Figure 1, left). Similarly, the LDH level increased significantly from 1 to 12 hours after LPS injection in the control group and stayed low in the DA group (Figure 1, middle). Although there was no such remarkable difference in the BUN level, the difference was significant at 1 hour after LPS injection (Figure 1, right).

**Figure 2:**
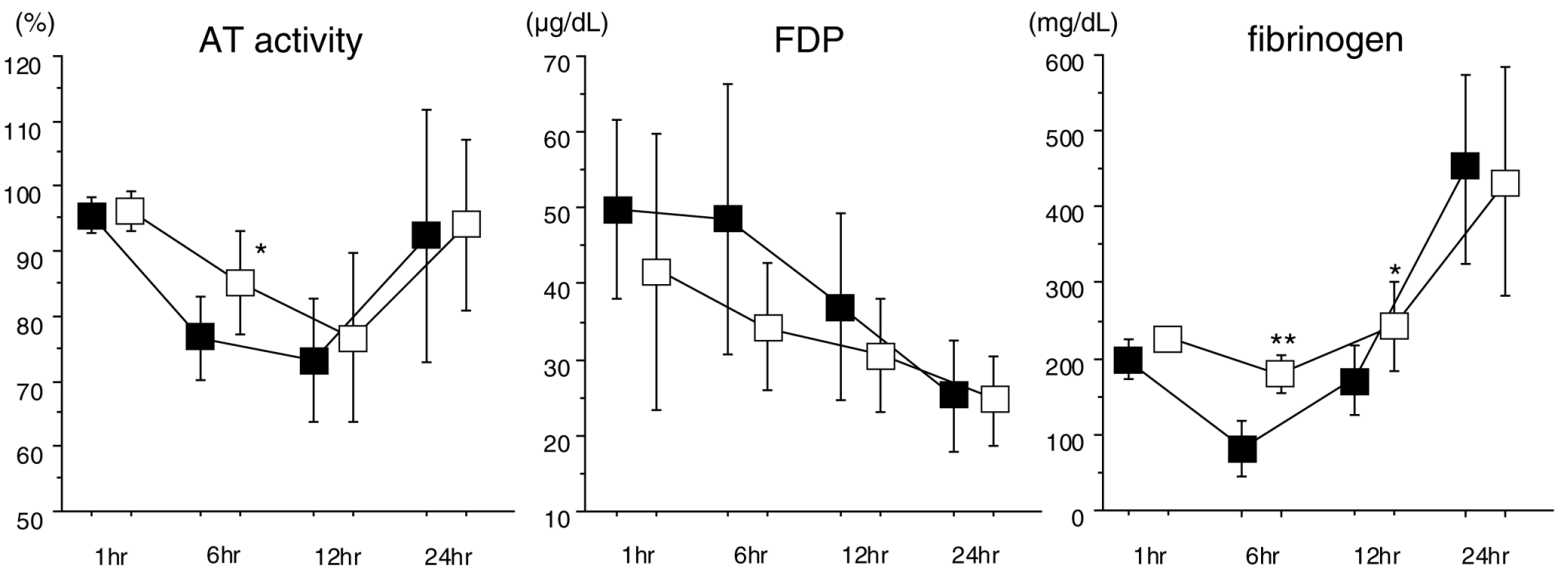
The changes in antithrombin (AT) activity, fibrin/fibrinogen degradation products (FDP), and fibrinogen after lipopolysaccharide (LPS) injection. AT activity decreased after LPS injection and recovered to the normal range at 24 hours in both groups. Although there was no significant difference in the bottom level, the difference was significant at 6 hours. The FDP level had already increased at 1 hour after LPS injection and decreased to 25 μg/dL in both groups at 24 hours; the difference was not significant during the time course. The fibrinogen level decreased after LPS injection and was lowest at 6 hours; the difference was significant at this time point (*P < 0.05, **P < 0.01) (■: control group, □: danaparoid sodium group, n = 8 in each group).

AT activity decreased gradually after LPS injection, and the level reached bottom at 12 hours in both groups. AT activity was significantly higher at 6 hours in the DA group (Figure 2, left). The FDP level had already increased at 1 hour after LPS injection and decreased to 25 μg/dL in both groups at 24 hours, and no significant difference was seen during the time course (Figure 2, middle). The fibrinogen level decreased after LPS injection and was lowest at 6 hours, and the significant difference was observed at this time point (Figure 2, right).

**Figure 3:**
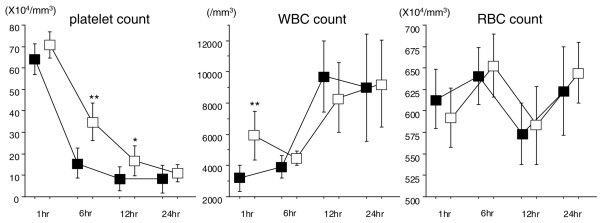
The changes of white blood cell (WBC), platelet, and red blood cell (RBC) counts after lipopolysaccharide (LPS) injection. The WBC count had already decreased at 1 hour after LPS injection and was significantly lower in the control group. The platelet count decreased after LPS injection and was lower in the control group at 6 and 12 hours after LPS injection. The RBC count stayed at similar levels, and no difference was observed between the groups (*P < 0.05, **P < 0.01) (■: control group, □: danaparoid sodium group, n = 8 in each group).

The WBC count had already decreased at 1 hour after LPS injection and was significantly higher in the DA group (Figure 3, left). The platelet count decreased over time after LPS injection and was maintained better in the DA group (Figure 3, middle). The RBC count stayed at a similar level, and there was no difference between the groups (Figure 3, right).

**Figure 4:**
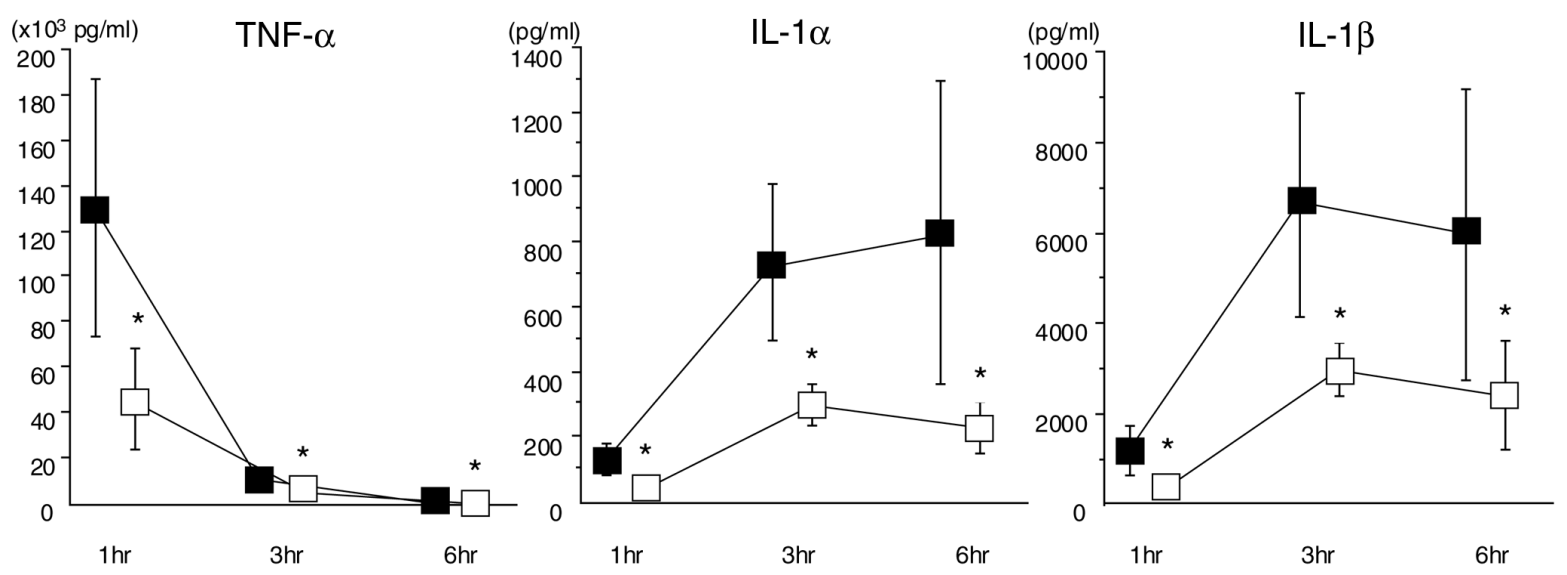
The changes of tumor necrosis factor-alpha (TNF-α), interleukin (IL)-1α, and IL-1β. The TNF-α level had already increased 1 hour after LPS injection and decreased over the time. The TNF-α level was suppressed in the danaparoid sodium (DA) group throughout the experimental period. Both IL-1α and IL-1β levels were significantly higher in the control group compared with the DA group (*P < 0.05) (■: control group, □: DA group, n = 5 in each group).

The TNF-α level elevated sharply and reached over 100,000 pg/mL in the control group but stayed at approximately one third of that level in the DA group (*P *< 0.05). The TNF-α level decreased rapidly thereafter in both groups, but the difference was still significant at 3 and 6 hours (*P *<0.05, respectively) (Figure 4, left). The changes of IL-1α and IL-1β showed a similar pattern. But the level was approximately 10 times higher in IL-1β and reached over 6,000 pg/mL in the control group at 3 hours. The levels of IL-1α and IL-1β in the DA group stayed at less than half of those of the control group throughout the experimental period, and the difference was significant (*P *<0.05, respectively) (Figure 4, middle and right).

**Figure 5:**
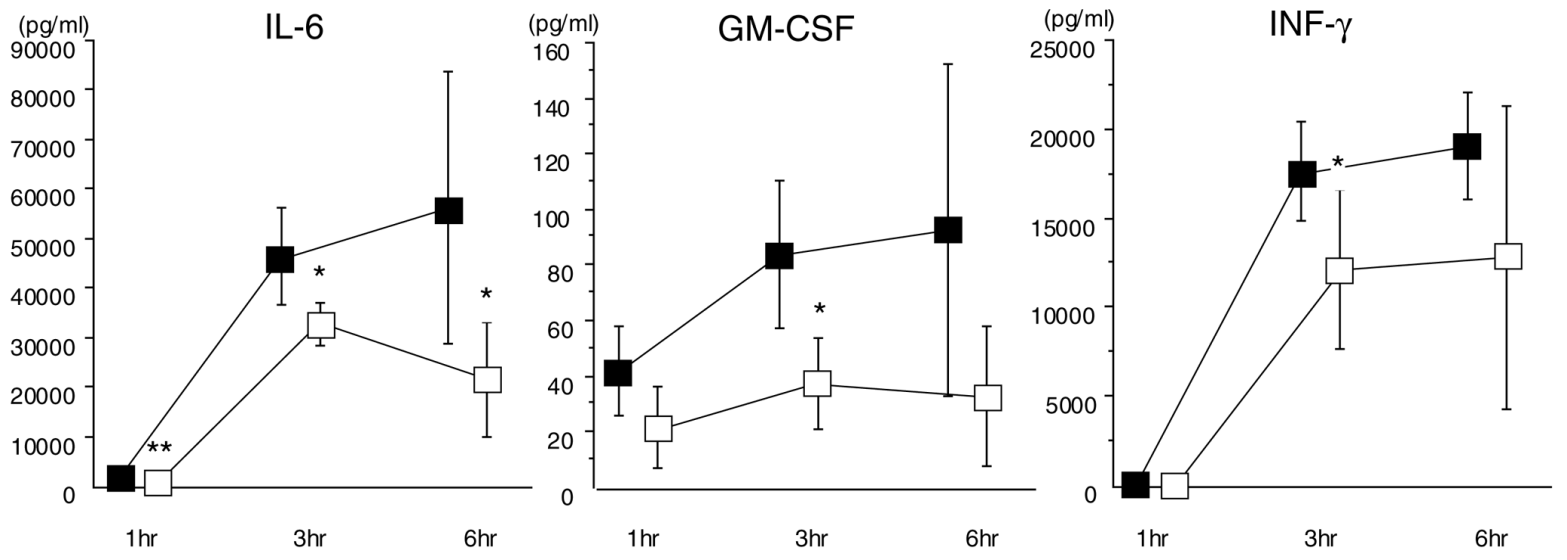
The changes of interleukin (IL)-6, granulocyte-macrophage colony-stimulating factor (GM-CSF), and interferon-gamma (INF-γ). The IL-6 level increased over the time in the control group and was significantly higher in the control group compared with the danaparoid sodium (DA) group at 1, 3, and 6 hours after lipopolysaccharide (LPS) injection. Both GM-CSF and INF-γ levels were higher in the control group throughout the experimental period, and the difference was significant at 3 hours after LPS injection (*P < 0.05, **P < 0.01) (■: control group, □: DA group, n = 5 in each group).

Significant elevation in IL-6 level was not recognized at 1 hour in either group. In the DA group, the level reached 32,807 ± 4,320 pg/mL and decreased thereafter. In contrast, the level increased over time and reached 56,191 ± 27,564 pg/mL at 6 hours in the control group, and the difference was significant at 1 hour (*P *< 0.01) and 3 and 6 hours (*P *< 0.05, respectively) (Figure 5, left). The change of GM-CSF was not remarkable and stayed at less than 100 pg/mL in both groups. However, the level was higher in the control group throughout the experiment and the difference was significant at 3 hours (*P *< 0.05) (Figure 5, middle). The INF-γ level was elevated at 3 hours after LPS injection and was higher in the control group (*P *< 0.05 at 3 hours) (Figure 5, right).

**Figure 6:**
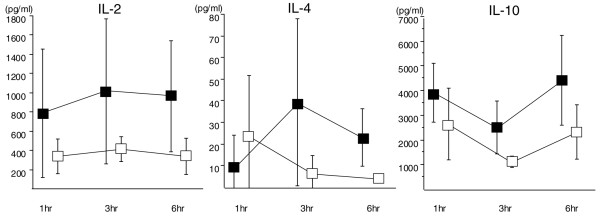
The changes of interleukin (IL)-2, IL-4, and IL-10. Although the control group showed higher IL-2, IL-4, and IL-10 levels, the difference was not significant throughout the time course (■: control group, □: danaparoid sodium group, n = 5 in each group).

IL-2 levels at 1 hour were 785.4 ± 669.5 pg/mL in the control group and 338.2 ± 178.4 pg/mL in the DA group. IL-2 was sustained at a higher level at 3 and 6 hours than at 1 hour in the control group, whereas the level stayed similar to that at 1 hour in the DA group. Although the IL-2 level in the DA group stayed at less than half of that in the control group, the difference was not statistically significant (Figure 6, left). The change of IL-4 was little in both groups and the level was less than 40 pg/mL throughout the experimental period (Figure 6, middle). IL-10 levels at 1 hour increased to 3,907 ± 1,188.3 pg/mL in the control group and 2,651.4 ± 1,458.3 pg/mL in the DA group. Although the level was higher in the control group at 3 and 6 hours, the difference was not significant at any time point (Figure 6, right).

## Discussion

The association of activated intravascular coagulation is widely recognized as a critical determinant of morbidity and mortality in the progression from systemic inflammatory response syndrome to multiple organ dysfunction syndrome during sepsis [10-12]. Although clinically overt disseminated intravascular coagulation (DIC) occurs in only 30% to 50% of septic patients, the activation of coagulation cascade is an early and universal response to the systemic infection [13-15]. Based on this knowledge, the anticoagulant therapy for sepsis is expected to reduce mortality and morbidity. Several animal and human studies have been performed and have suggested that some, but not all, of the anticoagulants have beneficial effects [16-18]. For example, activated protein C has been approved as the first drug for severe sepsis, and even heparin, which is more widely available and is commonly recommended to infuse in the treatment of DIC [13,19], modulates a wide array of responses to infection [20-24]. However, as summarized in a recent editorial in the *Journal of the American Medical Association *[25], the potential of heparin and its attractive usefulness for the treatment of sepsis have not been tested; therefore, the HETRASE (unfractionated heparin for treatment of sepsis) study, a randomized clinical trial testing low-dose continuous infusion of unfractionated heparin (500 U/hour for 7 days) as a complementary treatment for septic patients, is now being conducted.

HS is a heparin-like molecule that consists of linear polysaccharides comprised of repeating disaccharide units of uronic acid and *N-*substituted glucosamine [26]. HS is found ubiquitously expressed on cell surfaces and in extracellular compartments. HS *in vivo *is found covalently conjugated to specific core proteins as HSPGs and has been found to bind and regulate most of the key mediators of tissue injury and inflammation. Thus, HS is thought to coordinate the host response to infectious tissue injury [2,3]. The pathological function of HS in the pathogenesis of inflammatory diseases is not fully understood; however, HS is known to regulate molecular and cellular interactions relevant to inflammation.

In a former study, we demonstrated that DA effectively magnifies the anti-inflammatory effects of AT since it has relatively lower binding affinity for AT in comparison with unfractionated heparin [27]. In addition, we hypothesized that DA has beneficial effects for the treatment of sepsis by itself and then examined the effects of DA on organ dysfunction and inflammatory reaction in a rat LPS-challenged model.

The host responses during sepsis are mediated by various inflammatory factors. HS has been found to bind and regulate most of these key mediators. Recent studies demonstrated that externally administered DA can reduce the organ damage in an ischemia reperfusion model [28]. In contrast, Hollenstein and colleagues [29] reported that DA does not alter endotoxin-induced changes in the cytokine levels and activation of leukocytes. Therefore, the primary purpose of this study was to examine whether DA is efficacious for septic organ dysfunction. As a result, organ damage markers such as ALT and LDH were significantly improved by the treatment of DA from 1 to 12 hours after LPS infusion.

The dose of DA was fixed based on the dose-escalation study. The anti-Xa activity reached the effective range of 0.38 ± 0.31 IU at 1 hour after the infusion with 400 U/kg of DA. In regard to the pharmacokinetics, the elevated activity sharply decreased to 0.20 ± 0.15 and 0.13 ± 0.07 IU at 3 and 6 hours after DA infusion, respectively, and the activity returned to baseline thereafter. Consequently, AT activity and fibrinogen level showed the levels of AT activity and fibrinogen were higher in DA group compared to the control group at 6 hours. Although the difference was statistically significant in these markers, it was not impressive. Furthermore, the difference was not significant in FDP level. Therefore, we speculated that there should be other functions that may contribute to the attenuation of organ dysfunction.

TNF-α and IL-1 are proinflammatory cytokines that are elaborated by monocytes or macrophages and play pivotal roles in the development of organ dysfunction associated with sepsis. TNF-α induces organ damage by activating neutrophils and endothelial cells as well as coagulation abnormalities in patients with sepsis [30]. Thus, inhibition of TNF-α and IL-1 production as well as reduction of coagulation abnormalities might be critical for treating septic organ dysfunction. In addition to these changes of early mediators, other inflammatory cytokines such as IL-6, GM-CSF, and INF-γ were also lower in the DA group compared with the control group. In contrast to the suppression of proinflammatory cytokines, the levels of anti-inflammatory cytokines such as IL-4 and IL-10 did not differ in this experiment. We speculate that the changes of these cytokines may relate to the maintenance of the organ function. As for the regulation of cytokine levels by DA, to our knowledge there has been no publication other than this report.

With regard to the other possible mechanism of action, Harada and colleagues [28] demonstrated that DA enhanced the release in calcitonin-gene-related peptide (CGRP), a neuropeptide released from sensory neurons in rats subjected to ischemia reperfusion injury. CGRP ameliorates the organ damage through the increase of endothelial production of PGI_2 _(prostacyclin) by activating endothelial nitric oxide synthase and cyclooxygenase-1. Further study should be done to clarify the mechanism of this therapy.

## Conclusion

DA improves the organ dysfunction in LPS-challenged rats. Although the mechanism is not fully elucidated, not only the improvement of coagulation disorder but also the regulation of circulating levels of proinflammatory cytokines may play a role in the mechanism.

## Key messages

• Danaparoid sodium (DA) improves the organ dysfunction in lipopolysaccharide (LPS)-challenged rats.

• DA suppresses the proinflammatory cytokine levels in LPS-challenged rats.

## Abbreviations

ALT = alanine aminotransferase; AT = antithrombin; BUN = blood urea nitrogen; CGRP = calcitonin-gene-related peptide; DA = danaparoid sodium; DIC = disseminated intravascular coagulation; FDP = fibrin/fibrinogen degradation products; GM-CSF = granulocyte-macrophage colony-stimulating factor; HS = heparan sulfate; HSPG = heparan sulfate proteoglycan; IL = interleukin; INF-γ = interferon-gamma; LDH = lactate dehydrogenase; LPS = lipopolysaccharide; RBC = red blood cell; TNF-α = tumor necrosis factor-alpha; WBC = white blood cell.

## Competing interests

This work was financially supported by Organon International Inc. (Roseland, NJ, USA). The authors state that they have no other conflict of interest.

## Authors' contributions

TI designed the study, processed the data, and wrote the manuscript. TM performed the experiment and collected the data. Both authors read and approved the final manuscript.

## References

[B1] Meuleman DG (1992). Orgaran (Org 10172): its pharmacological profile in experimental models. Haemostasis.

[B2] Park PW, Reizes O, Bernfield M (2000). Cell surface heparan sulfate proteoglycans: selective regulators of ligand-receptor encounters. J Biol Chem.

[B3] Bernfield M, Götte M, Park PW, Reizes O, Fitzgerald ML, Lincecum J, Zako M (1999). Functions of cell surface heparan sulfate proteoglycans. Annu Rev Biochem.

[B4] Bartlett AH, Hayashida K, Park PW (2007). Molecular and cellular mechanisms of syndecans in tissue injury and inflammation. Mol Cells.

[B5] Alexopoulou AN, Multhaupt HA, Couchman JR (2007). Syndecans in wound healing, inflammation and vascular biology. Int J Biochem Cell Biol.

[B6] Kuschert GS, Coulin F, Power CA, Proudfoot AE, Hubbard RE, Hoogewerf AJ, Wells TN (1999). Glycosaminoglycans interact selectively with chemokines and modulate receptor binding and cellular responses. Biochemistry.

[B7] de Paz JL, Moseman EA, Noti C, Polito L, von Andrian UH, Seeberger PH (2007). Profiling heparin-chemokine interactions using synthetic tools. ACS Chem Biol.

[B8] Lyon M, Rushton G, Askari JA, Humphries MJ, Gallagher JT (2000). Elucidation of the structural features of heparan sulfate important for interaction with the Hep-2 domain of fibronectin. J Biol Chem.

[B9] Yu W, Woessner JF (2000). Heparan sulfate proteoglycans as extracellular docking molecules for matrilysin (matrix metalloproteinase 7). J Biol Chem.

[B10] Hotchkiss RS, Karl IE (2003). The pathophysiology and treatment of sepsis. N Engl J Med.

[B11] Opal SM, Esmon CT (2003). Bench-to-bedside review: functional relationships between coagulation and the innate immune response and their respective roles in the pathogenesis of sepsis. Crit Care.

[B12] Levi M, Ten Cate H (1999). Current Concepts. Disseminated intravascular coagulation. N Engl J Med.

[B13] Levi M, De Jonge E, Poll T Van Der, Ten Cate H (2000). Novel approaches to the management of disseminated intravascular coagulation. Crit Care Med.

[B14] Mavrommatis AC, Theodoridis T, Orfanidou A, Roussos C, Christopoulou-Kokkinou V, Zakynthinos S (2000). Coagulation system and platelets are fully activated in uncomplicated sepsis. Crit Care Med.

[B15] Opal S, Garber GE, LaRosa SP, Maki DG, Freebairn RC, Kinasewitz GT (2003). Systemic host responses in severe sepsis analyzed by causative microorganism and treatment effects of Drotrecogin Alfa (activated). Clin Infect Dis.

[B16] Choi G, Vlaar AP, Schouten M, Van't Veer C, Poll T van der, Levi M, Schultz MJ (2007). Natural anticoagulants limit lipopolysaccharide-induced pulmonary coagulation but not inflammation. Eur Respir J.

[B17] Kipnis E, Guery BP, Tournoys A, Leroy X, Robriquet L, Fialdes P, Neviere R, Fourrier F (2004). Massive alveolar thrombin activation in *Pseudomonas aeruginosa *– induced acute lung injury. Shock.

[B18] Slofstra SH, van 't Veer C, Buurman WA, Reitsma PH, ten Cate H, Spek CA (2005). Low molecular weight heparin attenuates multiple organ failure in a murine model of disseminated intravascular coagulation. Crit Care Med.

[B19] Ten Cate H (2000). Pathophysiology of disseminated intravascular coagulation in sepsis. Crit Care Med.

[B20] Tanaka T, Tsujinaka T, Kambayashi J, Higashiyama M, Yokota M, Sakon M, Mori T (1990). The effect of heparin on multiple organ failure and disseminated intravascular coagulation in a sepsis model. Thromb Res.

[B21] Meyer J, Cox CS, Herndon DN, Nakazawa H, Lentz CW, Traber LD (1993). Heparin in experimental hyperdinamic sepsis. Crit Care Med.

[B22] Boldt J, Papsdorf M, Piper SK, Rothe A, Hempelmann G (1999). Continuous heparinization and circulating adhesion molecules in the critically ill. Shock.

[B23] Pernerstorfer T, Hollenstein U, Hansen J-B, Knechtelsdorfer M, Stohlawetz P, Graninger W (1999). Heparin blunts endotoxin-induced coagulation activation. Circulation.

[B24] Derhaschnig U, Pernerstorfer T, Knechtelsdorfer M, Hollenstein U, Panzer S, Jilma B (2003). Evaluation of anti-inflammatory and antiadhesive effects of heparins in human endotoxemia. Crit Care Med.

[B25] Angus DC, Crowther MA (2003). Unraveling severe sepsis. Why did OPTIMIST fail and what's next?. JAMA.

[B26] Esko JD, Selleck SB (2002). Order out of chaos: assembly of ligand binding sites in heparan sulfate. Annu Rev Biochem.

[B27] Iba T, Kidokoro A, Fukunaga M, Nagakari K, Suda M, Yoshikawa S, Ida Y (2005). Antithrombin ameliorates endotoxin-induced organ dysfunction more efficiently when combined with danaparoid sodium than with unfractionated heparin. Intensive Care Med.

[B28] Harada N, Okajima K, Kohmura H, Uchiba M, Tomita T (2007). Danaparoid sodium reduces ischemia/reperfusion-induced liver injury in rats by attenuating inflammatory responses. Thromb Haemost.

[B29] Hollenstein UM, Pernerstorfer T, Homoncik M, Hansen JB, Finzen H, Handler S, Jilma B (2002). Effect of factor X inhibition on coagulation activation and cytokine induction in human systemic inflammation. J Infect Dis.

[B30] Okajima K (2001). Regulation of inflammatory responses by natural anticoagulants. Immunol Rev.

